# Mental Health Status of People with Multiple Sclerosis during the COVID-19 Pandemic

**DOI:** 10.3390/jcm11030576

**Published:** 2022-01-24

**Authors:** Maciej Wilski, Magdalena Koper, Jarosław Gabryelski, Waldemar Brola, Tomasz Tasiemski

**Affiliations:** 1 Department of Adapted Physical Activity, Poznań University of Physical Education, 61-871 Poznan, Poland; koper@awf.poznan.pl (M.K.); tasiemski@awf.poznan.pl (T.T.); 2 Division of Rehabilitation Engineering, Institute of Combustion Engines and Transport, Faculty of Machines and Transport, Poznan University of Technology, 60-965 Poznan, Poland; jaroslaw.gabryelski@put.poznan.pl; 3 Department of Neurology, Specialist Hospital in Końskie, Collegium Medicum, Jan Kochanowski University, 25-369 Kielce, Poland; wbrola@wp.pl

**Keywords:** mental health, multiple sclerosis, COVID-19, self-efficacy, health locus of control

## Abstract

Objective. This study assesses and compares the mental health status of people with multiple sclerosis (PwMS) in Poland during the second wave of the Coronavirus Disease 2019 (COVID-19) pandemic (November 2020) to a similar group whose mental health status was examined in November 2017. It also analyzed the psychological resources such as self-efficacy and health locus of control (HLC) and their relationship to mental health in both groups. Methods. Cross-sectional study included two groups of PwMS with 113 respondents each. The respondents completed the General Health Questionnaire-12 and questionnaires for assessing self-efficacy and HLC. The clinical and demographic data of participants were also collected. Results. No differences in mental health status were observed between the studied groups. A hierarchical regression model of the group studied in 2020 revealed that general self-efficacy (β = −0.21, *p* = 0.032), HLC—internal (β = −0.21, *p* = 0.035), and education (β = −0.18, *p* = 0.048) explained 18% of the variance in the mental health of PwMS, whereas according to the model of the group assessed in 2017 self-efficacy (β = –0.31, *p* < 0.001), HLC—chance (β = 0.45, *p* < 0.001), and HLC—internal (β = −0.37, *p* < 0.001) explained 48% of the variance. Conclusions. Study results suggest that the pandemic and the related lockdown had no effect on the mental health status of PwMS. At the same time, it was noted that well known determinants of mental health such as self-efficacy and HLC seemed to retain their prominent role for mental functioning in the pandemic.

## 1. Introduction

The Coronavirus Disease 2019 (COVID-19) pandemic infection first occurred in China in December 2019 and quickly spread throughout the world. It was eventually declared as a global pandemic on March 11, 2020. As of November 2021, more than 267 million confirmed COVID-19 cases have been identified and over 5 million deaths were reported worldwide. In Poland, severe acute respiratory syndrome coronavirus 2 (SARS-CoV-2) infection was first officially reported at the beginning of March 2020. Before 19 September 2020, the number of daily new cases did not exceed 1000. However, the second wave of the pandemic hit the country a few weeks later and with much greater power (with more than 30,000 new cases a day, toward the end of November) [1]. According to the Eurostat data, Poland had the highest rate of excess deaths compared to other European Union member states in 2020. In November 2020, the excess death rate in Poland reached 97%, with nearly twice as many deaths as usual in the month. Considering the epidemiological situation of the country, the government imposed a restrictive lockdown on October 24.

At that stage of the pandemic, the effect of COVID-19 on immune diseases such as multiple sclerosis (MS) was unknown. It was believed that people with MS (PwMS) may be at an increased risk of SARS-CoV-2 infection and complications due to their immunocompromised state, MS-related comorbidities, and use of immunosuppressive therapies [2,3]. Their feeling of insecurity was exacerbated by questions about the course of COVID-19 in PwMS, the effect of COVID-19 on the course of MS, restrictions in access to health care, misinformation, lack of vaccines, and concern about vaccination in PwMS. Moreover, PwMS had to experience the social effects of lockdown such as limited social interactions, quarantine, changes in daily routine, loss of income, fear of losing a job, uncertainty about the future, loss of loved ones, and fear of contracting COVID-19. All these factors have been shown by early studies to have had a negative impact on mental health or exacerbate preexisting psychiatric challenges, in both the general population and people with chronic disorders [4,5,6,7]. In addition, regardless of the pandemic, the prevalence of depression and anxiety is greater among PwMS compared to the general population [8], and so PwMS have a higher risk of developing mental health disorders.

Studies assessing the relationship between the pandemic and the mental state of PwMS have reported varied results. While some have associated the COVID-19 pandemic with a higher level of negative mental health indicators (such as depression or anxiety) [3,9,10,11,12,13], the results of some studies do not confirm this relationship [2,14,15,16,17]. This discrepancy in results may be due to the use of different methodologies in studies: some studies were qualitative and were based on an interview, and some did not include a control group or included healthy individuals as controls. Furthermore, most of the studies available in the literature were conducted in the Italian population. Another reason for the inconsistent results may be a limited analysis of the other possible factors that may significantly influence the functioning of an individual during a pandemic. For example, lockdown and the resulting social environment may affect an individual’s perception of potential opportunities to act in the context of his/her own health. The imposed restrictions and the prevailing sense of uncertainty may contribute to reducing the psychological resources essential for good mental functioning, such as the sense of self-efficacy or health locus of control (HLC). Previous research results have clearly indicated that these personal resources enable PwMS to effectively cope with the disease or difficulties of daily life and manage the treatment process on their own, which directly translates into their mental functioning [18,19,20,21].

Self-efficacy refers to an individual’s belief that he/she can accomplish a specific situation or a task and reflects an individual’s confidence in coping with a difficult, demanding, or limiting situation [22]. During the COVID-19 pandemic, several studies reported that higher general self-efficacy was related to less mental health problems in several populations [23,24]. There are no data available for PwMS.

The HLC has three categories: internal HLC refers to individuals’ belief that their own behaviors affect their health status, whereas external HLC refers to individuals’ belief that their health is attributed to other people—mainly medical professionals—or chance variables such as luck and fate. The latest research shows that HLC plays an important role in moderating the association of COVID-19 stress and mental distress in general population [25]. Researchers indicate that the locus of control shifted significantly from internal to external during the pandemic in the studied groups, which is explained by increased feelings of powerlessness in the early phase of the pandemic [26,27]. So far, no research in this area has been performed in the PwMS.

Taking the above into account, there is a need to assess the mental health status of PwMS during the pandemic. Our study also included a group of PwMS, with comparable demographic and clinical variables, as a control group, whose mental health status and psychological resources were examined 3 years earlier in the same period of the year (November 2017). Within this context, we aimed to (1) compare mental health status of PwMS studied in 2020 and control group studied in 2017; (2) compare the level of psychological resources such as self-efficacy and HLC in study and control group; (3) evaluate the extent to which socio-demographic, clinical, and psychological factors are associated with mental health in both groups.

## 2. Materials and Methods

### 2.1. Participants and Procedures

This cross-sectional study included two groups of PwMS who were enrolled in November 2017 and 2020 with the help of the Department of Neurology at Specialist Hospital in Konskie, Poland. Each group consisted of 113 PwMS recruited using the same criteria and principles. The inclusion criteria for the study were as follows: (1) diagnosis of definite MS by a neurologist according to the revised McDonald criteria [28]; (2) no relapse in the last 30 days before the enrollment; (3) absence of cognitive problems and/or psychiatric disorders as confirmed by a neurologist; (4) absence of any concomitant disease. An additional criterion set for the recruitment of the 2020 group was the current lack of symptoms and a diagnosis of COVID-19. The participants were recruited by a research team member during their control visit at the clinic. After providing verbal instructions and explaining the purpose of the study, the individuals were asked if they were interested to participate. Those who were willing to take part in the study were asked to read and complete a questionnaire in a quiet room allocated specifically for this purpose. The study adhered to the ethical standards of the Declaration of Helsinki, and written informed consent was obtained from all the participants prior to the study. After receiving the set of forms, the questionnaire was immediately checked for completeness, and the respondents were asked to fill all potential gaps. The STROBE checklist for cross-sectional studies was used for reporting the study. Ethical approval was obtained from the Bioethical Commission of Poznan University of Medical Sciences.

### 2.2. Measures

All research tools used in the study were standardized and validated in many previous studies on various groups of patients, including PwMS. The mental health of the participants, which was the dependent variable of this study, was evaluated using the Polish version of General Health Questionnaire-12. This questionnaire was developed and validated by Goldberg and Williams [29] and adapted by Makowska and Merecz [30]. It was designed for use in nonpsychiatric medical consultations by neurologists to detect mental functioning changes in patients. GHQ was previously used to assess mental health in the MS population [19,31,32]. The questionnaire has 12 questions for assessing the general level of happiness, perceived stress, the experience of depressive and anxiety symptoms, and sleep disturbance over the last 4 weeks. The examinees should choose among four Likert-type responses on the scale, ranging from “*less than usual*” to “*much more than usual*.” The total score ranges from 0 to 36, with higher scores indicating a worse psychological condition. The Cronbach’s alpha value for the study samples was 0.82.

The Multidimensional Health Locus of Control Scale (MHLC) was first developed by Wallston, Strudler Wallston, and DeVellis [33], and then adapted and validated to Polish by Juczyński [34]. The MHLC has 18 items that assess participants’ perception of how much control they have over their medical decisions, with scores assigned to two categories: internal HLC and external HLC. The external HLC comprises two subscales: power of others HLC (the belief that one’s health is influenced by others, for example, physicians or other healthcare professionals) and chance HLC (the belief that one’s health depends on chance, luck, or fate). Each of these subscales consists of items with a 6-point Likert-type response scale, ranging from 1 (*strongly disagree*) to 6 (*strongly agree*), with higher scores indicating a more intense HLC in a given dimension. MHLC has been used in previous studies with a group of people with MS [35,36,37]. The internal consistency rates in this study for internal, power of others, and chance HLC scales were 0.72, 0.80, and 0.68, respectively.

The self-efficacy of PwMS was measured using the Generalized Self-Efficacy Scale (GSES) [38], which was designed to assess the strength of one’s belief in his/her own abilities to respond to novel or difficult situations. The GSES was adapted to polish by Juczynski [34]. The scale consists of 10 items with a 4-point scale, ranging from 1 (*not at all true*) to 4 (*exactly true*), with higher scores indicating higher self-efficacy. GSES has been used in previous studies with a group of people with MS [39,40,41].The internal consistency rate of GSES in the study samples amounted to 0.96.

The clinical and demographic data of participants were collected using a standardized questionnaire. The clinical variables assessed in the study were as follows: illness duration, MS subtype, and overall MS severity, evaluated using the Expanded Disability Status Scale (EDSS) [42]. The EDSS is extensively used to assess the progression of disease and neurological disability. The scale spans from EDSS 0 (normal neurological examination) to EDSS 10 (death from MS) in half-point increments starting from EDSS 1. The EDSS scores and MS subtype of participants were determined by a neurologist. Gender, age, educational level, place of residence, employment, monthly income of one family member, and marital status were the sociodemographic variables analyzed in the study.

### 2.3. Data Analysis

#### 2.3.1. Matching Samples and Preliminary Analysis

By propensity score matching, the control group (2017) was formed by matching PwMS who were examined in November 2020 (during the second wave of the COVID-19 epidemic) with the PwMS examined in 2017 using the 1:1 nearest neighbor method. The following variables were considered for matching: age, gender, level of education, and EDSS scores. After matching, a preliminary analysis was performed by comparing demographic, socioeconomic, illness, and psychological variables between the two groups using Mann–Whitney test (*Z*), Pearson’s chi-square test, and maximum likelihood chi-square test.

Finally, a well-balanced cohort of 113 paired PwMS (assessed in 2020 and 2017) was analyzed (Table 1). In terms of matching variables, the control group (2017) did not differ from the study group (2020); however, the extracted group of 113 respondents differed from the total sample of 382 PwMS included in 2017. The mean age of the extracted participants was 43.1 years (SD = 10.3) and that of the total sample was 46.4 years (SD = 11.9), which showed a significant difference (*Z* = 2.75, *p* = 0.006). The mean EDSS score of the control group was 3.31 (SD = 0.81) and that of the total sample was 4.35 (SD = 1.68), and this difference was also significant (*Z* = 5.90, *p* < 0.001). No significant differences were found between the extracted sample and the total sample studied in 2017 with regard to gender (*p* = 0.409) and level of education (*p* = 0.119).

#### 2.3.2. Main Analysis

First, the data collected for the study were screened and checked against the assumptions of the regression analysis. Then, the presence and power of relationships between all independent variables (demographic, socioeconomic, illness, and psychological) and the dependent variable (mental health outcomes) was determined using the Mann–Whitney test (*Z*), Kruskal–Wallis test (*H*), and Spearman correlation (*r*_s_), in order to verify whether these variables should be controlled in future analyses. Finally, two separate hierarchical multiple regression analyses were performed, with mental health scores as dependent variables for the 2020 group (study group) and 2017 group (control group). Only the factors that were significantly associated with mental health outcome were included in the models. The level of statistical significance for the inclusion of independent variables in multiple regression models was set at *p* < 0.05. All statistical analyses were carried out in Statistica software, version 4.0.67 (StatSoft Polska, www.statsoft.pl).

## 3. Results

### 3.1. Study Participants

The mean age of both 2020 (*n* = 113) and 2017 (*n* = 113) groups was just above 40 years. About two-thirds of the participants in the groups were female, and more than 60% of the participants had secondary or higher education (Table 1). The disability status was almost the same in the study and control group (EDSS = 3.31). The majority of participants in the groups lived in towns, and were married. Significant differences were found in employment status between the groups (*p* = 0.011). The post hoc analysis revealed that 57.5% of PwMS were employed in 2020 and 45.1% were employed in 2017 (*p* = 0.004). Similarly, significant differences were noted between the groups in terms of the monthly income of one family member (*p* < 0.001). The post hoc analysis indicated that more PwMS in the 2020 group had a monthly income of above 2000 PLN (*p* < 0.001), and an income between 1500 PLN and 2000 PLN (*p* = 0.013) in comparison to the control group studied in 2017. By contrast, a lower number of PwMS had a monthly income below 1000 PLN in 2020 than in 2017 (*p* < 0.001). The mean time elapsed from the MS diagnosis was 7.4 years in the 2020 group and 9.4 years in the 2017 group, but this difference was not significant. Significant differences were noted in the subtype of MS (*p* < 0.001). The post hoc analysis showed that more PwMS in 2020 had relapsing-remitting MS (82.3%) than in 2017 (54.0%), and this difference was significant (*p* < 0.001). On the other hand, less PwMS had secondary progressive MS in 2020 (4.4%) than in 2017 (17.7%), and this difference was also significant (*p* = 0.001).

### 3.2. Comparison of Mental Health and Psychological Factors in a Study and Control Group

No significant differences were identified between the groups with regard to mental health and general self-efficacy (Table 2). Significant differences were found with regard to the three forms of HLC (internal (*p* < 0.001), powerful others (*p* = 0.008), and chance (*p* < 0.001)), which are the three psychological variables of the study, between the analyzed groups. In all cases, higher mean scores were observed in the control group (2017) in comparison to the study group (2020). The Cohen’s d effect size was used to evaluate the clinical value of the differences in the outcomes of both groups. The Cohen’s d effect for internal HLC was 0.603, for chance HLC 0.657 and for powerful others HLC 0.334.

### 3.3. Associations of Independent Variables with Mental Health in Study and Control Groups

The coefficients of association between all independent variables and mental health scores in PwMS (study and control groups) are presented in Table 3. For the study group (2020), seven factors were found to be significantly associated with mental health scores i.e., gender (*p* = 0.024), level of education (*p* = 0.022), employment status (*p* = 0.043), time from diagnosis (*p* = 0.007), diagnosed MS subtype (*p* = 0.018), general self-efficacy (*p* < 0.001)*,* HLC—internal (*p* = 0.009). In the case of the control group (2017), significant association with mental health scores were found for nine factors i.e., age (*p* < 0.001), gender (*p* = 0.049), marital status (*p* = 0.046), level of education (*p* < 0.001), employment status (*p* = 0.002), monthly income of one family member (*p* < 0.001), general self-efficacy (*p* < 0.001), HLC—internal (*p* < 0.001), and HLC—chance (*p* = 0.003).

### 3.4. Hierarchical Multiple Regression

Two separate hierarchical multiple regression analyses were carried out using mental health scores as a dependent variable (Table 4).

In the first regression model of the 2020 study group, three out of seven factors related to mental health, namely general self-efficacy (*β* = −0.21, *p* = 0.032), HLC—internal (*β* = −0.21, *p* = 0.035), and education (*β* = −0.18, *p* = 0.048), which were significantly associated with the dependent variable, were included. In the regression analysis, all these three factors were identified as significant correlates of mental health in MS. Persons with vocational education were found to have significantly lower mental health compared to those with secondary (*p* = 0.013) and higher education (*p* = 0.002). Although the model reached the threshold of statistical significance, it explained only 18% of the variance in mental health in MS (*R*^2^ = 0.18, *F*(3.109) = 7.94, *p* < 0.001).

In the second regression model of the 2017 control group, three out of nine factors related to mental health, namely self-efficacy (*β* = −0.31, *p* < 0.001), HLC—chance (*β* = 0.45, *p* < 0.001), and HLC—internal (*β* = −0.37, *p* < 0.001), which were significantly associated with the dependent variable, were included. All of them were identified as significant correlates of mental health in MS. The model was significant and explained 48% of the variance in mental health in MS (*R*^2^ = 0.48, *F*(3.109) = 33.32, *p* < 0.001).

## 4. Discussion

A comparison of mental health parameters of two similar PwMS groups in Poland—one studied before the pandemic and the other during the second wave of the pandemic—showed no differences between the groups in mental health status. Thus, the results support the thesis, and, also, those research results indicating that there is no relationship between the pandemic and deterioration of mental health in PwMS. This situation may be similar to that of the general population. Although numerous reports have shown that the mental health of the general population has been drastically affected, along with a higher prevalence of depression, anxiety, and stress during a lockdown and increased cases of suicides, the first major meta-analysis of longitudinal studies and natural experiments showed that lockdowns do not have uniformly detrimental effects on mental health and that most people are psychologically resilient to their effects [43]. Moreover, evidence suggests that MS experience immunizes an individual, to some extent, against the negative emotional consequences of a pandemic. For example, Chiaravalloti and colleagues [2] reported that PwMS wanted to safeguard themselves early in the pandemic due to their heightened risk of infection and subjective emotions of vulnerability. As they were careful in following safety precautions, their attempts of self-protection may have boosted their comfort level, reducing anxiety and depression. In addition, because of their illness and associated disability, PwMS more often experience social isolation, and may, therefore, find it easier to adapt to lockdown restrictions [44,45]. Another explanation is living in fear of one’s own health due to COVID-19. PwMS have dealt with this feeling since their diagnosis of MS, which is an unpredictable, uncertain, and uncontrollable disease, and thus can easier deal with the discomfort that comes with it [16,46,47]. Finally, based on their previous experience coping with a chronic condition and attacks, PwMS may have evolved more effective ways to manage stress during the acute COVID-19 outbreak [16]. All these assumptions certainly require more in-depth research in order to arrive at final conclusions.

Regarding the second goal of this study, the analyzed groups did not differ in terms of general self-efficacy. Thus, our results do not support those researchers who indicate that COVID-19 and lockdown significantly decreased the sense of self-efficacy in the general population [48,49]. Perhaps, in PwMS, self-efficacy beliefs are more stable, developed in coping with MS and more resistant to changing external conditions. Certainly, in order to provide an unambiguous answer, it is worth undertaking more in-depth research in this direction. Additionally, both regression analysis of the present study revealed the importance of self-efficacy in mental health which confirms the results of other studies [50,51], including those undertaken during the pandemic [45].

Significant differences were noted in HLC between the studied groups. For all three forms of HLC (internal, powerful others, and chance), higher mean scores were observed for the group studied in 2017 in comparison to the 2020 group. This is partly confirmed by reports from the general population indicating subjective loss of control during the pandemic, as a reaction to extensive restrictions and the unpredictability of the pandemic’s dynamics [27]. However, these studies while pointing to a decrease in the internal HLC, at the same time indicated an increase in the external HLC, which we did not observe in our studies. Perhaps, a pandemic generally lowers the sense of control over one’s health (internal or external) in PwMS, but this conclusion should be confirmed by more targeted, population-extended studies. Undoubtedly, the unidirectional changes in HLC dimensions constitute another argument for the thesis that HLC dimensions are independent of each other and require a separate investigation [52].

Regarding the relationship of HLC with mental health, an association between internal HLC and better mental health was found in both studied groups. This result is further evidence of the positive importance of the internal HLC in shaping the well-being of PwMS [19,36], and the pandemic did not contribute to changes in this regard.

Other variables included in the regression analysis indicated that education was significant correlate of mental health during the pandemic. More precisely, persons with vocational education were characterized by significantly lower mental health compared to those with secondary and higher education. It can be speculated that a higher level of knowledge and awareness of the course of COVID-19 can support mental functioning in PwMS, which suggests the need for further research in this area.

This study has several limitations. First, due to its cross-sectional nature, the study did not allow drawing any conclusions regarding the direction of causal relationships between the analyzed variables. The second limitation is the difficulty in generalizing the results of the study to the PwMS population in other countries. The course of the COVID-19 pandemic and lockdown restrictions differed in individual countries, which certainly may have had an impact on the mental well-being of PwMS. Moreover, it was unclear whether the respondents had previously suffered from COVID-19. It should be mentioned that the history of this disease and its severity may significantly influence the mental health of individuals. Third, people suffering from cognitive disorders, which are a frequent consequence of MS, were excluded from the study. Cognitive impairments were only assessed based on a subjective neurological examination, but were not confirmed by any objective tool, and so this exclusion criterion is unreliable. Finally, the study did not identify any difficulties or barriers directly experienced by respondents as a result of the pandemic. Their detailed identification and analysis of their relationships with mental health should be the goal of further research on the mental functioning of PwMS in a pandemic.

Despite these limitations, the findings of this study demonstrate that the pandemic and the related lockdown were not associated with mental health status of PwMS in Poland. The results contradict the popular belief that the pandemic has had a negative effect on the mental health of people. At the same time, it was noted that well known determinants of mental health, such as self-efficacy and HLC seemed to retain their prominent role for mental functioning in the pandemic. However, while self-efficacy did not change, all categories of HLC decreased, compared to the pre-pandemic period. Further research should focus on clarifying the role of HLC, in order to help mental health specialists appropriately guide their patients during the pandemic.

## Figures and Tables

**Table 1:** Socio-demographic and clinical characteristic of persons with MS studied in 2020 and control group studied in 2017.

	Persons with MS				
Socio-Demographic, Clinical, and Psychological Characteristics	Study Group (*n* = 113)		Control Group (*n* = 113)		*p* Value
	Matching variables				
Age (years ± SD)	42.2 ± 9.3		43.1 ± 10.3		0.603 ^a^
Gender *n* (%)					0.890 ^b^
Male	41	(36.3)	42	(37.2)	
Female	72	(63.7)	71	(62.8)	
Education *n* (%)					0.879 ^c^
Primary	2	(1.8)	1	(0.9)	
Vocational	35	(31.0)	39	(34.5)	
Secondary	40	(35.4)	37	(32.7)	
Higher	36	(31.9)	36	(31.9)	
Disability EDSS (mean ± SD)	3.31 ± 0.9		3.31 ± 0.8		0.916 ^a^
		Other	variables		
Place of living *n* (%)					0.179 ^b^
Town	59	(52.2)	69	(61.1)	
Village	54	(47.9)	44	(38.9)	
Marital status *n* (%)					0.147 ^b^
Single/separated/divorced	20	(17.7)	22	(19.5)	
Married/relationship	93	(82.3)	91	(80.5)	
Employment *n* (%)					**0.002** ^b^
Employed	65	(57.5)	51	(45.1)	
Unemployed/retired	48	(42.5)	62	(54.9)	
Monthly income to one family member *n* (%)					**<0.001** ^b^
Less than 1000 PLN	17	(15.1)	55	(48.6)	
1000–1500 PLN	34	(30.1)	34	(30.1)	
1500–2000 PLN	31	(27.4)	16	(14.2)	
More than 2000 PLN	31	(27.4)	8	(7.1)	
Time from diagnosis (years ± SD)	7.7 ± 5.3		9.4 ± 7.5		0.166 ^a^
Diagnosed type of MS *n* (%)					**<0.001** ^c^
Relapsing-remitting (RRMS)	93	(82.3)	61	(54.0)	
Primary progressive (PPMS)	12	(10.6)	22	(19.5)	
Secondary progressive (SPMS)	5	(4.4)	20	(17.7)	
Progressive-relapsing (PRMS)	2	(1.8)	4	(3.5)	
Do not know (DKMS)	1	(0.9)	6	(5.3)	

Note: ^a^ Mann-Whitney test; ^b^ Pearson’s chi-square test; ^c^ maximum likelihood chi-square test; bold values indicate statistically significant differences.

**Table 2:** Comparison of mental health and psychological factors of persons with MS studied in 2020 and control group studied in 2017.

Psychological Factors (Mean ± SD)	Persons with MS		*p* Value
	Study Group (*n* = 113)	Control Group (*n* = 113)	
Mental health	29.24 ± 4.00	28.72 ± 5.45	0.710 ^a^
General self-efficacy	31.05 ± 5.30	29.83 ± 5.20	0.079 ^a^
Health locus of control—internal	21.89 ± 4.30	24.50 ± 4.35	**<0.001** ^a^
Health locus of control—others	24.18 ± 4.46	25.72 ± 4.76	**0.008** ^a^
Health locus of control—chance	21.88 ± 4.17	24.92 ± 4.63	**<0.001** ^a^

Note: ^a^ Mann–Whitney test; bold values indicate statistically significant differences.

**Table 3:** Associations between selected independent variables and mental health in persons with MS studied in 2020 and control group studied in 2017.

	Mental Health			
	Study Group (*n* = 113)		Control Group (*n* = 113)	
Independent Variables	Statistical Test	*p* Value	Statistical Test	*p* Value
Demographic factors				
Age	r_s_ = 0.05	0.570	r_s_ = 0.32	**<0.001**
Gender	Z = 2.25	**0.024**	Z = 1.97	**0.049**
Marital status	Z = −0.53	0.593	Z = −1.99	**0.046**
Education	H = 9.67	**0.022**	H = 16.69	**<0.001**
Place of living	Z = 1.72	0.086	Z = 1.41	0.159
Socioeconomic factors				
Employment	Z = −2.02	**0.043**	Z = −3.12	**0.002**
Monthly income	H = 3.24	0.356	H = 18.65	**<0.001**
Illness factors				
Disability EDSS	r_s_ = 0.10	0.302	r_s_ = 0.01	0.910
Time from diagnosis	r_s_ = 0.25	**0.007**	r_s_ = 0.12	0.205
Diagnosed type of MS	H = 11.91	**0.018**	H = 5.56	0.234
Psychological factors				
General self-efficacy	r_s_ = −0.32	**<0.001**	r_s_ = −0.48	**<0.001**
Health locus of control—internal	r_s_ = −0.24	**0.009**	r_s_ = −0.39	**<0.001**
Health locus of control—others	r_s_ = −0.15	0.123	r_s_ = 0.03	0.719
Health locus of control—chance	r_s_ = 0.04	0.711	r_s_ = 0.27	**0.003**

Note: Spearman correlation (r_s_); Mann–Whitney test (Z); Kruskal–Wallis test (H); bold values indicate statistically significant associations.

**Table 4:** Regression analysis predicting mental health in persons with MS studied in 2020 and control group studied in 2017.

Variable	*R* ^2^	*β*	*F*	*p* Value
Model 1 Study group 2020	0.18		7.94	**<0.001**
General self-efficacy		−0.21		**0.032**
Health locus of control—internal		−0.21		**0.035**
Education		−0.18		**0.048**
Model 2 Control group 2017	0.48		33.32	**<0.001**
General self-efficacy		−0.31		**<0.001**
Health locus of control—chance		0.45		**<0.001**
Health locus of control—internal		−0.37		**<0.001**

Bold values indicate statistically significant associations.

## Data Availability

The data that support the findings of this study are openly available in RepOD at https://doi.org/10.18150/GLZKEP (accessed on 15 January 2022).
